# Electrochemotherapy for feline lingual squamous cell carcinoma: a case report of functional preservation as a palliative approach

**DOI:** 10.3389/fvets.2026.1850098

**Published:** 2026-07-20

**Authors:** Makoto Akiyoshi, Masaharu Hisasue

**Affiliations:** 1 Akiyoshi Animal Clinic, Yamato, Kanagawa, Japan; 2 Laboratory of Small Animal Internal Medicine, School of Veterinary Medicine, Azabu University, Sagamihara, Kanagawa, Japan

**Keywords:** bleomycin, electrochemotherapy, feline, lingual squamous cell carcinoma, occult tumor spread, oral squamous cell carcinoma, quality of life, total glosso-mandibulectomy

## Abstract

A 14-year-old spayed female Persian cat with lingual feline oral squamous cell carcinoma (FOSCC) was treated with bleomycin-based electrochemotherapy (ECT), resulting in sustained preservation of oral function and quality of life despite advanced disease. At presentation, the cat exhibited severe weight loss, oral pain, and reduced voluntary food intake. ECT was performed using intravenous bleomycin (20 mg/m^2^) followed by electric pulse delivery via an eight-needle electrode array. Following treatment, marked tumor regression was observed, accompanied by substantial reduction in oral pain, restoration of voluntary feeding, and body weight gain from 1.7 kg to 2.6 kg during the treatment period. Voluntary eating and drinking were preserved throughout the clinical course until the terminal stage. Repeated examinations under general anesthesia revealed no gross abnormalities in the larynx or tonsils. The cat died due to renal failure unrelated to tumor progression. Postmortem histopathology revealed extensive SCC infiltration involving the tongue base, tonsils, and epiglottis despite the absence of clinical abnormalities during life. This case demonstrates that ECT can provide meaningful palliation, including preservation of oral function and quality of life, in cats with biologically advanced lingual FOSCC. In addition, the marked discrepancy between clinical findings and histopathologic tumor extent suggests the possibility of clinically inapparent regional spread, which may have important implications for staging and treatment planning. These findings also suggest that ECT may preserve superficial functional structures while allowing progression of submucosal tumor components.

## Introduction

Feline oral squamous cell carcinoma (FOSCC) is a common oral malignancy associated with poor prognosis and severe impairment of quality of life, with reported median survival times often less than 2 months in untreated cases (1–5). Lingual squamous cell carcinoma (SCC) represents approximately 10–20% of FOSCC and frequently involves the ventral midline of the tongue, extending toward the tongue base and sublingual tissues (6). These tumors are typically associated with severe oral pain, dysphagia, and progressive weight loss, leading to substantial impairment of quality of life.

Radical surgical approaches, including mandibulectomy and total glosso-mandibulectomy (TGM), have been described in selected cases; however, accurate delineation of tumor extent remains challenging, and complete excision may be difficult to achieve (7–11). In addition, such procedures are associated with significant morbidity and may not always be acceptable to owners. Radiation therapy and chemotherapy have shown limited and often short-lived efficacy (12–15). Given these limitations, treatment strategies that prioritize preservation of oral function and quality of life may be particularly relevant in cats with lingual FOSCC. In this context, preservation of oral function may be more clinically meaningful than aggressive attempts at complete tumor eradication.

Electrochemotherapy (ECT) enhances intracellular uptake of chemotherapeutic agents via electroporation and has demonstrated efficacy in various feline tumors, including cutaneous SCC (16–19). However, its application in lingual FOSCC remains poorly characterized, particularly with respect to functional outcomes.

This case not only describes the clinical course of lingual SCC treated with ECT but also provides insight into the potential discrepancy between functional preservation and underlying tumor progression. We hypothesize that ECT may allow maintenance of oral function despite ongoing submucosal tumor spread, which may remain clinically undetectable.

In addition, this case highlights the presence of clinically inapparent regional tumor spread, which may have important implications for clinical assessment and treatment planning. To our knowledge, this is one of the first reports demonstrating a clear dissociation between functional preservation and extensive occult regional spread under ECT in feline lingual SCC.

## Case description

**Figure 1:**
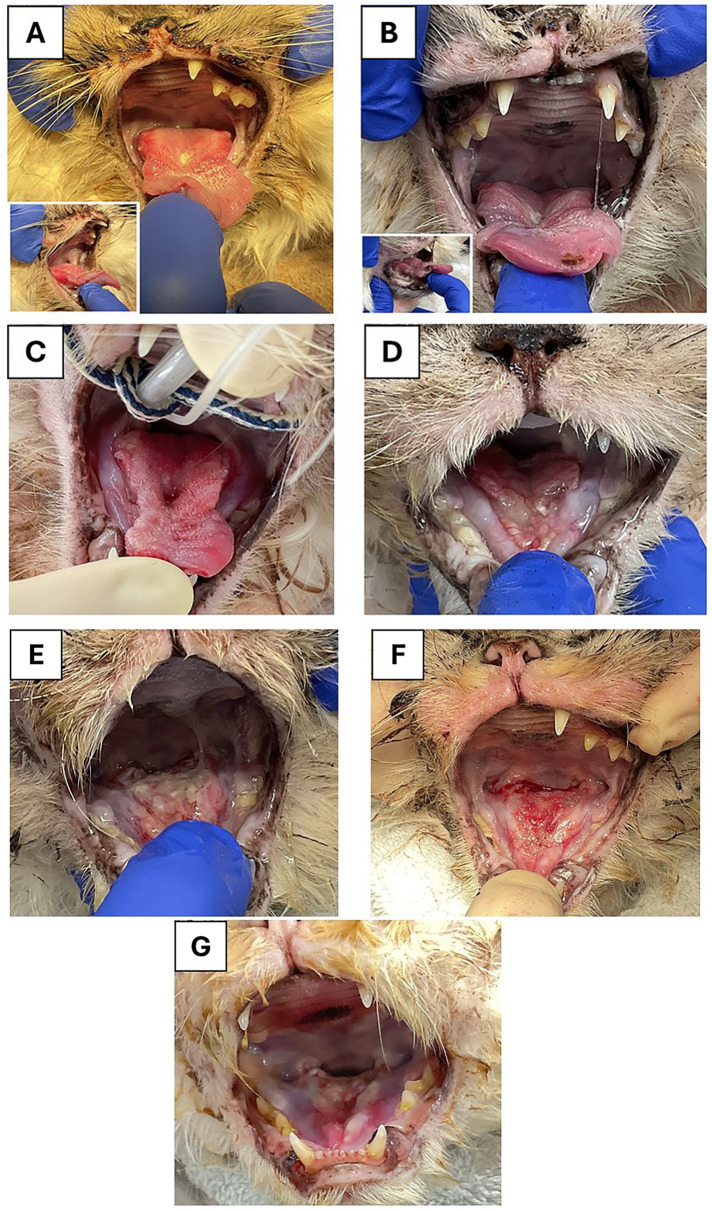
Macroscopic progression of lingual squamous cell carcinoma during electrochemotherapy treatment. **(A)** Day 1, **(B)** Day 10, **(C)** Day 31, **(D)** Day 59, **(E)** Day 97, **(F)** Day 101, and **(G)** Day 120. Progressive tumor reduction is observed. Although tumor shrinkage was observed macroscopically, the contribution of treatment-induced necrosis cannot be excluded. This limitation should be considered when interpreting the apparent tumor response. No gross abnormalities were detected in the larynx, tonsils, or soft palate throughout the clinical course.

A 14-year-old spayed female Persian cat (body weight 1.7 kg; previous weight 2.8 kg) was referred for evaluation of a one-month history of hyporexia, ptyalism, and oral bleeding. Physical examination revealed severe dehydration, cachexia, and a painful mass involving the entire length of the tongue from the apex to the base (Figure 1A). Although no enlargement of the retropharyngeal or other regional head lymph nodes was detected on palpation or ultrasonographic examination, advanced imaging such as computed tomography (CT) was not performed. This limitation should be considered when interpreting the extent of disease and the conclusions of this report and may have affected the accuracy of staging. Therefore, the possibility of subclinical regional involvement cannot be excluded, and staging accuracy in this case was limited.

Cytology of the lingual mass was consistent with SCC. Fine-needle aspiration of both mandibular lymph nodes revealed reactive hyperplasia. Fine-needle aspiration cytology was performed only for the bilateral mandibular lymph nodes, as these were the only lymph nodes accessible for sampling. Other regional lymph nodes, including the retropharyngeal lymph nodes, were not amenable to cytological evaluation, which represents a limitation in the assessment of regional lymph node status. Thoracic and abdominal radiographs and abdominal ultrasonography showed no evidence of distant metastasis. However, advanced imaging such as computed tomography (CT) was not performed, and therefore the presence of subclinical metastatic disease cannot be excluded. Hematologic evaluation identified non-regenerative anemia (PCV 22%) and azotemia (BUN 69 mg/dL; creatinine 2.9 mg/dL), consistent with IRIS stage 3 chronic kidney disease. At presentation, the cat was severely cachectic, with a body condition score (BCS) of 1/9.

**Figure 2:**
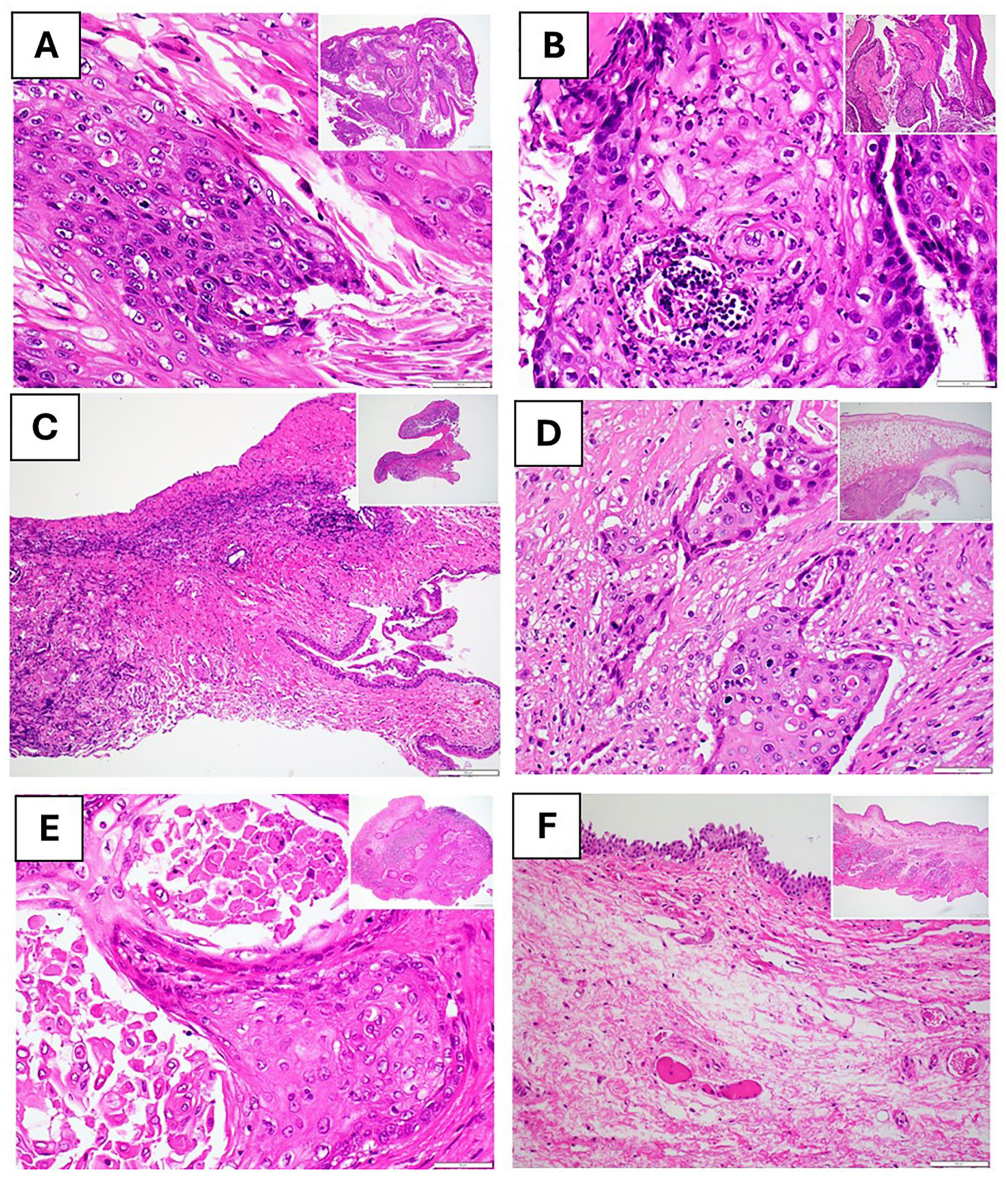
Histopathological findings. **(A)** Shows squamous cell carcinoma (SCC) of the tongue prior to initiation of electrochemotherapy (ECT). **(B)** Shows SCC of the tongue at the third ECT session, with no significant changes observed compared to before ECT initiation. **(C)** Shows the pathological findings of the tonsils at the third ECT session, with no evidence of SCC infiltration. **(D–F)** are histopathological findings at autopsy, showing the larynx, tonsils, and soft palate, respectively. SCC infiltration was observed in the larynx and tonsils, but not in the soft palate. Note that the surface structure of the mucosa was preserved in both the larynx and tonsils.

Incisional biopsy confirmed invasive SCC (Figure 2A). Due to advanced age, pre-existing chronic kidney disease, and owner preference, radical surgery and radiation therapy were declined. Electrochemotherapy (ECT) using intravenous bleomycin was elected (16–19). Bleomycin was selected due to its established use in ECT protocols (16–19) and its favorable efficacy-to-toxicity profile in feline patients. Intravenous administration was selected due to the diffuse involvement of the entire tongue. The drug was administered intravenously at a dose of 20 mg/m^2^, followed by electric pulse delivery 8 min later, according to standard protocols (16–19). ECT was performed under general anesthesia using a veterinary electroporation device (ELECTROVET EZ; LEROY Biotech, France) with an 8-needle electrode array. Bleomycin (20 mg/m^2^ IV) was administered, and electric pulses were delivered 8 min later (1,000 V/cm; 500 V). The injected volume of bleomycin was calculated based on body surface area and diluted in saline for intravenous administration. Electric pulses consisted of eight square-wave pulses of 100 μs duration at a frequency of 5,000 Hz. The electrode array was applied sequentially in overlapping positions to ensure complete coverage of the tumor area. Needle insertion depth was adjusted to match the thickness of the tongue tissue, ensuring adequate electric field distribution throughout the lesion. Electrodes were applied circumferentially to achieve adequate tumor coverage while avoiding major vascular structures. Due to the diffuse induration involving the entire tongue, treatment was applied to the whole tongue. Electroporation was intentionally limited to the tongue, and surrounding tissues were not included in the electric field to preserve function. Electrode application patterns and the number of electrode applications were adjusted based on tumor size and anatomical constraints of the tongue to balance treatment efficacy and preservation of function. Three ECT sessions were performed on days 3, 31, and 81. The number of electrode applications was progressively reduced across sessions (50, 40, and 25 applications), reflecting treatment adaptation based on tumor volume reduction. At the first session, direct visualization of the larynx and tonsils revealed no abnormalities, and an esophagostomy tube was placed for nutritional support. The initial UNESP-Botucatu Feline Pain Scale (UFEPS) score was 17 (20). By day 10, tumor size had decreased, bleeding was reduced, and the UFEPS score improved to 7 (Figure 1B). Supportive care consisted of the application of a fentanyl transdermal patch following each ECT session. No additional medications such as NSAIDs or corticosteroids were administered. Although RECIST criteria were not strictly applicable due to the irregular shape and anatomical location of the lesion, tumor response was assessed consistently based on serial macroscopic evaluation and clinical improvement. Body weight increased to 1.9 kg, and activity improved.

By day 31, voluntary food intake resumed, tumor regression continued, and the UFEPS score improved to 2 (Figure 1C). A second ECT session was performed at that time. By day 59, the mass had further regressed, the UFEPS score decreased to 1, and body weight increased to 2.6 kg (Figure 1D). The restoration of voluntary feeding represented a major clinical turning point, as the cat had previously been unable to maintain adequate oral intake.

On day 81, mild tonsillar changes prompted biopsy, which revealed inflammatory changes without evidence of malignancy (Figure 2C), while the lingual lesion remained consistent with SCC (Figure 2B). A third ECT session was performed. On day 97, continued tumor regression and preservation of oral function were observed (Figure 1E).

On day 101, the cat developed upper respiratory signs. Endoscopic examination revealed no gross abnormalities in the larynx, nasopharynx, or tonsils aside from the primary lingual lesion (Figure 1F). Thoracic radiographs (three-view) were performed monthly, and no radiographic evidence suggestive of pulmonary metastasis was identified during the follow-up period. PCR restong confirmed viral infection. Supportive managemnet, including nasal irrigation and administration of a Huaier-derived preparation (Animmune®, HACHI Co., Ltd., Japan; TPG-1), was initiated as adjunctive therapy. Clinical signs subsequently resolved; however, the relative contribution of each intervention remains unclear.

Throughout the treatment period, voluntary eating and drinking were preserved, and oral function was maintained until the terminal stage despite continued supportive nutritional management. Although progressive tongue atrophy was observed macroscopically, the cat remained able to drink water independently and ingest soft food, including paste-type diets, according to owner observations. No severe adverse events directly attributable to ECT were observed during the treatment period. Complete blood counts were also monitored regularly and did not reveal clinically significant abnormalities. Renal parameters were monitored monthly and remained stable throughout the treatment period, with a marked increase observed only at the terminal stage. On day 120, the cat developed severe uremia and died despite supportive care. No clinical deterioration attributable to oral disease was observed at that time (Figure 1G). Postmortem histopathological examination revealed SCC infiltration involving the tongue base, tonsils, and epiglottis despite the absence of gross abnormalities during life (Figures 2D–F).

## Discussion

The most clinically relevant outcome in this case was the sustained preservation of oral function and quality of life following ECT. Lingual SCC is typically associated with severe oral pain, dysphagia, and rapid deterioration in nutritional status, making maintenance of voluntary feeding a critical determinant of patient welfare (1–4). Recent comprehensive reviews of feline oral SCC have emphasized its aggressive nature and the limited survival benefit associated with current treatment modalities, supporting the role of palliative strategies focused on quality of life (11). In this cat, ECT was associated with marked reduction in pain, restoration of voluntary food intake, and substantial weight gain, indicating a meaningful improvement in quality of life. This case illustrates that treatment success in lingual FOSCC should not be defined solely by tumor reduction, but also by preservation of essential functions such as voluntary feeding. Although tumor reduction was observed, the contribution of supportive care to the improvement in body condition cannot be entirely excluded. However, given the limited supportive interventions, ECT likely played a major role in tumor control.

ECT in the tongue presents unique technical challenges due to the highly vascular and muscular nature of this organ. High-intensity electric field exposure in highly vascularized and muscular tissues such as the tongue may result in edema, hemorrhage, or tissue necrosis, potentially compromising function. In this case, careful electrode placement and controlled delivery of electric pulses allowed effective tumor treatment without inducing complete tissue sloughing. The tongue is composed primarily of skeletal muscle and is richly vascularized, which may influence both drug distribution and tissue response to electroporation. In the present case, complete tissue sloughing did not occur. Macroscopic tumor reduction observed in this case may partially reflect treatment-induced necrosis rather than true tumor regression, which represents a limitation in interpreting response. Instead, gradual tumor regression accompanied by tissue contraction was observed. No severe adverse events directly attributable to ECT were observed. Follow-up monitoring, including monthly thoracic radiographs and hematologic and biochemical analyses, revealed no clinically significant abnormalities or evidence of treatment-related toxicity, and no progression of pre-existing renal disease was detected. This finding may reflect a balance between effective tumor cell kill and preservation of structural integrity. ECT enhances cytotoxic drug uptake through transient membrane permeabilization (16–19), but may also allow preservation of extracellular matrix and overall tissue architecture when excessive ischemic damage is avoided. The use of a multi-needle electrode array may have contributed to a more uniform electric field distribution across the lesion, reducing the risk of localized overexposure. Previous preclinical and human studies have suggested that electric field distribution and electrode geometry are important determinants of ECT efficacy and tissue preservation, particularly in anatomically complex regions (21–23). In addition, adjustment of treatment parameters according to tumor response, including reduction in the number of electrode applications over successive sessions, may have helped to limit cumulative tissue damage while maintaining therapeutic efficacy. These observations suggest that individualized ECT protocols, taking into account both tumor burden and anatomical characteristics, may be important for optimizing functional outcomes in lingual tumors. These findings are consistent with previous reports demonstrating the feasibility of ECT in feline tumors, although application in lingual SCC remains limited, highlighting the need for further investigation in anatomically complex sites (16–19).

From a clinical perspective, this case supports the use of ECT as a function-preserving palliative treatment in selected patients with lingual FOSCC, particularly when radical surgery is declined, contraindicated, or unlikely to achieve complete oncologic control. Previous reports have suggested that the application of electrochemotherapy in lingual or oral tumors should be approached with caution due to the risk of functional impairment and treatment-related complications in this anatomically and functionally critical organ (16, 17). However, in the present case, ECT was successfully applied without compromising essential oral functions, resulting in sustained voluntary feeding and improved quality of life. This finding suggests that, when carefully performed, ECT may be a feasible and clinically valuable option even in lingual SCC, despite prior concerns. In such cases, treatment strategies that prioritize quality of life may represent a rational and proportionate approach. Similar functional preservation following ECT has been reported in previous veterinary studies involving oral or cutaneous tumors (16–19), supporting its role as a palliative treatment modality in selected cases. Although reports specifically describing ECT in feline oral tumors are limited, previous studies involving feline cutaneous and nasal planum SCC have demonstrated favorable clinical responses (16–19). In addition to the favorable functional outcome, this case revealed a marked discrepancy between clinical findings and actual tumor extent. Postmortem findings, although not available during clinical management, provide important insight into the discrepancy between clinical assessment and actual tumor extent (Figures 2D–F). Tonsillar evaluation was limited during the clinical course, and earlier detection of infiltration cannot be excluded. This finding suggests that lingual FOSCC may spread along submucosal or regional tissue planes without producing overt macroscopic changes.

This clinically inapparent regional extension has important implications for staging and treatment planning. Surgical approaches such as TGM rely on accurate delineation of tumor margins; however, occult tumor spread may limit the ability to achieve complete excision even when adjacent structures appear normal. The ability to achieve adequate surgical margins may be influenced by subclinical tumor spread; however, this observation should be interpreted with caution, as comprehensive staging, including advanced imaging, was not performed in this case. Furthermore, imaging modalities such as CT may underestimate microscopic or submucosal disease (3, 6, 8, 9). The ability to achieve adequate surgical margins may be influenced by the presence of subclinical tumor spread; however, this case does not allow definitive conclusions due to the lack of comprehensive staging. These findings highlight the need for caution when interpreting apparently localized disease in lingual FOSCC. One possible explanation for this discrepancy is that tumor spread in lingual SCC may occur predominantly along submucosal planes without disrupting the superficial mucosal architecture. As a result, clinically apparent findings may underestimate the true extent of disease. In this context, ECT may preferentially affect accessible tumor components while having limited impact on deeper or anatomically concealed tumor regions, potentially explaining the coexistence of functional preservation and histopathologic progression. Importantly, the present findings indicate that the clinical benefits of ECT, particularly in terms of functional preservation, may still be achieved even in cases with biologically advanced or occultly spreading disease. This observation is consistent with previous reports emphasizing the clinical relevance of palliative strategies focused on quality of life in FOSCC (11).

To the best of our knowledge, this is one of the first reports describing the clinical application of ECT specifically in feline lingual SCC. While ECT has been reported in feline cutaneous and nasal planum SCC (16–19), its use in the oral cavity—particularly in anatomically complex and functionally critical structures such as the tongue—has been approached with caution due to the potential risk of treatment-related morbidity. In the context of FOSCC, various palliative treatment options have been described, including radiation therapy, tyrosine kinase inhibitors such as toceranib, and bisphosphonates (4, 11–15). Reported median survival times for cats with FOSCC treated with palliative radiation therapy or toceranib are approximately 92–123 days (4, 12), although outcomes vary according to tumor location, treatment protocol, and concurrent therapies. In the present case, survival time was 120 days, which was within the range of previously reported palliative approaches. Importantly, voluntary feeding and drinking were maintained until the terminal stage, suggesting that ECT may provide clinically meaningful palliation by preserving oral function rather than prolonging survival alone. Notably, this case suggests a dissociation between functional outcome and underlying tumor progression, as substantial preservation of oral function was achieved despite histopathologically confirmed extensive regional tumor spread. This finding suggests that, in selected cases, ECT may represent a clinically relevant palliative option, particularly when preservation of essential functions is prioritized over complete tumor eradication. Furthermore, although advanced imaging such as CT was not performed, postmortem findings underscored the potential for clinically inapparent tumor extension, highlighting the limitations of clinical assessment alone.

The present case has several limitations, including its single-case design, absence of advanced imaging, and inability to determine the precise timing of regional tumor spread. In addition, the relative contribution of supportive care to the observed clinical improvement cannot be fully excluded. Adjunctive therapies were administered for concurrent conditions; however, these were not considered to have influenced tumor response or the preservation of oral function observed in this case. Nevertheless, the combination of sustained functional improvement and postmortem confirmation of extensive occult disease provides valuable insight into both therapeutic outcomes and tumor biology. This dissociation may have important implications for clinical decision-making, particularly when treatment response is assessed based on functional improvement alone.

## Conclusion

This case demonstrates that bleomycin-based ECT can provide meaningful palliation in cats with lingual SCC, including substantial pain reduction and preservation of oral function and quality of life. ECT may therefore represent a proportionate, function-preserving treatment option in biologically advanced cases where curative-intent therapy is not feasible. These findings support the potential applicability of ECT in lingual SCC, even in the presence of concerns regarding functional impairment, provided that treatment is carefully planned and executed.

In addition, this report highlights that lingual FOSCC may exhibit biologically extensive regional spread that remains clinically inapparent, complicating accurate assessment of tumor extent and potentially influencing the effectiveness of surgical intervention. Consideration of both functional outcomes and the possibility of occult disease may be essential when selecting treatment strategies in these patients. These findings highlight the need for caution when relying solely on clinical assessment to determine tumor extent and suggest that treatment strategies should consider the possibility of occult disease even in cases with preserved function. Further investigation in larger cohorts is warranted to determine the generalizability of these findings.

## Data Availability

The original contributions presented in the study are included in the article/supplementary material, further inquiries can be directed to the corresponding authors.
